# Understanding the influencing mechanism of users’ participation in live streaming shopping: A socio-technical perspective

**DOI:** 10.3389/fpsyg.2022.1082981

**Published:** 2023-01-13

**Authors:** Xueyan Dong, Xu Liu, Xuan Xiao

**Affiliations:** ^1^School of Management, Northwestern Polytechnical University, Xi'an, China; ^2^School of Management, Guangzhou University, Guangzhou, China

**Keywords:** live streaming, socio-technical perspective, IT afordance, attraction, cognitive assimilation, live streaming participation

## Abstract

**Introduction:**

In live streaming shopping, exploring the influencing mechanism of consumers’ participation is an important prerequi site for understanding consumer behavior in social commerce activities. The purpose of this study is to explore the relationship betw een technological and social factors (visibility, media richness, guidance shopping and real-time interactivity) in live streaming shop ping and consumers’ purchase intention. The mediating roles of attraction and cognitive assimilation were also examined.

**Methods:**

This study collected 425 pieces of data through questionnaire survey. The structural equation model is established based on S-O-R frame. The hypothesis is tested by structural equation model.

**Results:**

Our study found that that real-time interactive and media richness positively affect attraction; visibility, guidance shopping and media richness positive affect cognitive assimilation; cognitive assimilation and attraction positive affect consumers’ purchase intention; cognitive assimilation and attraction play a partial mediating role in the influence of technology and social factors on cons umers’ purchase intention of live streaming shopping.

**Discussion:**

From the perspective of socio-technical, this study explores the influence mechanism of different influencing factors on consumers’ purchase intention in live streaming shopping. This study expands the application of IT affordance theory in the context of live streaming shopping, and reveals the mediating role of attraction and cognitive assimilation between social, technological factors and consumers’ purchase intention.

## 1. Introduction

Live streaming shopping provides consumers with a more intuitive shopping experience based on its forceful interaction, which demonstrates a strong potential as a new form of social business (Sun et al., 2019). According to the survey data of China Internet Investigation Report, until December 2020, the number of online shopping users in China has reached 782 million. In 2020, the sales of live streaming shopping reached $6 billion, which is two times the number of sales in the same period of 2019 [China Internet Network Information Center (CNNIC), 2021]. Especially, due to the impact of COVID-19, many real industries have been greatly hit; however, live streaming shopping has grown against the trend, indicating that live streaming shopping has become a new force to boost economic development. Different from traditional e-commerce, live streaming shopping creates a scene of real-time display and instant two-way interaction, which greatly meets the user’s multidimensional needs of the shopping experience (Xu et al., 2020). Research shows that the visual presence in live streaming shopping helps consumers feel real and makes purchase decisions easier (Wongkitrungrueng and Assarut, 2020). Therefore, live streaming has become an important marketing tool for online platforms (e.g., social networking sites, virtual communities, and short video platforms), integrated with different industries to form “live +” embedded in consumers’ daily lives, and it has become the dominant scenario actions in the digital transformation of enterprises.

Compared with traditional e-commerce, live streaming shopping has developed into a unique business model with its own distinct features. First, real-time interaction between buyers and sellers can greatly enhance information transparency. Customers can interact with the streamer in real time through barrage questioning during any live streaming shopping period, it not only can shorten the perceived distance between buyers and sellers but also can effectively reduce customers’ uncertainty about products, as well as greatly eliminate customers’ perceived risks caused by online virtual context. As a result, it can increase the trust extent between buyers and sellers (Wongkitrungrueng, 2018). Second, as a new way of social networking, live streaming has a more abundant and diversified means of communication than traditional social networking does. Thereby, it can not only meet customers’ consumption needs but also can expand their entertainment needs (Busalim and Hussin, 2016). Finally, in the process of live streaming shopping, consumers can express their opinions and understand others through the interactive screenplay, praise, and comments. Accordingly, consumers’ purchase decisions-making is rather reasonable.

The practice of live streaming shopping is increasingly popular; however, research on this streaming is still in its early stage. Previous studies paid limited attention to exploring the impact mechanism of live streaming on users’ purchase intention (Chen and Lin, 2018). Wongkitrungrueng and Assarut’s study shows that live streaming can help promote consumer engagement (Wongkitrungrueng, 2018). Furthermore, most of the existing studies follow the research paradigm (e.g., driven factors) of traditional e-commerce to social commerce (Xu et al., 2020; Zhang et al., 2020). Xu et al. (2020) research shows that the interaction between streamers and consumers in the live streaming environment can stimulate consumers to produce different consumption behaviors. From the perspective of IT affordance, Sun et al. (2019) found that the technological features in live streaming shopping have a positive impact on consumers’ purchase intention. Existing research has shown that the building of strong and weak ties between buyers and sellers can help consumers to make purchase decisions (Dong and Wang, 2018), but we still know little about how the relationship works in the live streaming shopping context. In addition, the existing research mainly focused on a single point of view to explore the factors that affect users’ live streaming participation (e.g., trust issues, technical aspects, and online reviews; Ou et al., 2014; Li et al., 2020; Stsiampkouskaya et al., 2021); however, these studies cannot provide an integrated framework for understanding the reality of live streaming from a whole contextualized view. Thus, it has greatly hindered the academic innovative exploration of this novel phenomenon (Dong and Wang, 2018), since live streaming shopping is significantly different from other commerce in terms of technical functions, audience groups, and interactive ways (Floh and Madlberger, 2013).

Against the backdrop of these issues, we argue that there is a need to understand the contextualized factors driving consumers to make a purchase in live streaming and explore the specific impact mechanism that how these factors affect users’ purchase intention. To achieve this goal, we draw literature on social commerce and IT affordance and adopt a sociotechnical perspective to construct a contextualized model for the following three reasons. First, live streaming shopping is under the scope of social commerce, which is a prevalent socio-technical phenomenon, and the socio-technical perspective provides us with the opportunity to capture both social and technical elements simultaneously in framing and investigating technology-related societal issues (Li et al., 2021). Second, the socio-technical perspective, integrating the technical design with the social environment to cause goal-oriented behavior, is particularly well consistent with this study’s investigation of user’s intention to use live steaming to further make sure the goals of IT function to social purchases (Wong et al., 2021). In addition, the context of live steaming shopping merits new interaction insights regarding how the IT functionalities enable consumers to achieve social and shopping goals by shaping their cognition state, and this fits well our theoretical investigation.

The rest of this study is structured as follows. First, we review the related literature and provide a theoretical foundation. Then, based on the Stimulus-Organism-Response (S-O-R) framework, we build our research model from three levels: live streaming as a technology platform, live streaming as an online social context, and interplay of the technological functions and the social situation of live streaming. Next, we describe our research design and results. Finally, we discuss the study’s contributions, implications, and limitations, as well as future research. Our work is expected to shed light on theory development in this emerging live streaming research area and help practitioners understand how the desirable socio-technical system shapes consumers’ live streaming purchase intention.

## 2. Theoretical development

### 2.1. Live streaming shopping

Live streaming shopping represents the completion of mobile payment and online trading activities, while e-commerce activities perform trading through the network of live streaming platforms (Sun et al., 2019). It mainly includes a real-time live streaming platform, which provides a live environment for customers. Through live streaming technology and live streaming infrastructure, customers’ needs for interaction, entertainment, and shopping are met (Xu et al., 2020; Liu et al., 2022). From the point of view of information technology, live streaming is a way of releasing information by producing and releasing information on the spot. Through live streaming, the information publisher (streamer) can immediately send images and sounds to other locations, and users can receive information in real time (Chen and Lin, 2018). With the development in the vertical field, tourism, education, entertainment, games, and other industries have begun to adopt live streaming technology as a new development method. Compared to other live streaming industries, live streaming shopping has become an emerging representative industry with its speed and scale of development (Wang, 2019). With the emergence of a large number of streamers, the phenomenon of live streaming delivery quickly caught on the Internet and attracted widespread attention (Gong et al., 2022). Live streaming shopping is a new way of shopping; this new way allows consumers and sellers to interact in real time (Yang et al., 2022). Consumers can not only gain product information directly through the streamer’s introduction but also gain a better understanding of other users’ attitudes through the barrage and comments (Lu and Chen, 2021). At the same time, consumers can obtain shopping coupons or special goods by playing games and interactive lottery in the process of watching live streaming. This form of more entertaining shopping enables consumers to meet the enjoyment demand beyond the actual demand and stimulates the users to have purchase intention (Lim and Ayyagari, 2018). In traditional e-commerce activities, buyers and sellers do not know each other enough, which greatly affects the establishment of the trust relationship between buyers and sellers. On the contrary, in live streaming shopping, buyers and sellers interact between themselves to build strong emotional ties, which can help consumers eliminate the uncertainty generated by online shopping (Guo et al., 2021). To understand the particularity of live streaming shopping, this study summarizes the differences between live streaming shopping and other traditional shopping models, as shown in Table 1. The business model of live streaming shopping is fragmented, diversified, and fun-seeking. In the era of the digital economy, the emergence of live streaming shopping meets people’s online shopping needs more effectively.

**Table 1:** Comparison between live streaming shopping and other shopping models.

Shopping way	Content presenting	Feature	Example
Live streaming shopping	Real-time explanation + interaction + purchase	Bullet interaction, Instant comments, Convenient information consultation, Strong entertainment	Taobao live streaming, Douyin live streaming
Short video ecommerce	Browse + purchase	Interactive admiration, Instant comment, Strong entertainment, Concise video content, Concentrated commodity information	Micro video, Watermelon video, Volcano video
Traditional platform e-commerce	Browse + purchase	Rich commodity information, Information update lag, Communication inconvenience	Tian Mao mall, JD Mall, Amazon
Traditional offline shopping guide	Real-time explanation + accompany + purchase	Experience, Time consuming, Product information collection difficult	Wal-Mart, Carrefour and Hualian

### 2.2. Stimulus-organism-response model

The Stimulus-Organism-Response model (S-O-R) includes three parts: stimuli from the internal and external environment, the state of the organism, and their behavioral responses (Mehrabian and Russell, 1974). There are three key elements in this framework, namely stimulus, organism, and response. Stimuli can trigger individual emotional and cognitive processes that lead to the response of individual behavior (Donovan, 1994). The S-O-R model reveals the whole process of individual behavior, which is widely used in research studies of consumer behavior and online purchase (Chan et al., 2017; Xu et al. 2020). Zhang et al. (2014) applied the S-O-R model to the research on social commerce and explored the influence of technical characteristics (stimulus) in social commerce on customers’ virtual experience (organism) and customers’ participation intention (response); customers’ experience determines their final participation intention and is also influenced by the characteristics of social commerce. The stimulus represents the factors that attract the attention of the consumer, the organism represents the internal state of the consumer, and the reaction is the result of the response to the stimulus and the change in the internal state (Chan et al., 2017).

In the research on live streaming shopping, researchers apply the S-O-R model to explore the relationship between stimuli in the live streaming environment, consumers’ cognitive and emotional states, and resulting behaviors. For example, Xu et al. (2020) found that stimuli in the live streaming environment have a direct impact on the internal state of consumers and then affect consumer behavior. In the context of live streaming shopping, the S-O-R model well meets the needs of our study. First, this model can well summarize the environmental stimuli (such as technical features and social features of live streaming) in live streaming shopping in this study, thus researchers can summarize relevant influencing factors from a richer and more comprehensive perspective (Donovan 1994; Chan et al., 2017). Second, the model can help us observe the impact of environmental stimuli in live shopping on the internal states (cognitive and emotional states) of consumers, and how they ultimately influence consumer behavior. The core content of the S-O-R model is that environmental stimuli have an impact on the internal states and behaviors of individuals. Therefore, we believe that the adoption of this model can help us more clearly understand the impact of environmental stimuli in live streaming shopping on the internal state and behavior of consumers.

### 2.3. IT affordance

The affordance theory originated from the field of ecological psychology. It describes the phenomenon that the subject produces a certain behavior based on the perception of environmental characteristics (Gibson, 1978; Leonardi, 2011). This theory has gradually been widely used in the field of information systems and social psychology. In terms of the affordance definition, it means that the environment has different attributes, which could affect the way users achieve their goals in a specific context (Markus and Silver, 2008). Strong et al. (2014) believes that affordance is provided by technology or environment and focuses more on the possibility provided by the environment for individuals, which requires the interaction between the environment and individuals. Since Gibson (1978) proposed the idea of affordance, it has been improved and developed by many scholars in the field of ecological psychology and information systems, and many categories have been derived, such as perceived affordance, true affordance (Norman, 2002), and technological affordance (Markus and Silver, 2008). In reference to IT functionalities, IT affordance presents the possibility that the technology subject could provide a specific user group with specific technical features to achieve their goals (Treem and Leonardi, 2013). For years, IT affordance has been increasingly used in explaining how users and IT artifacts relate to each other. The notion of IT affordance has slightly different nuanced meanings and its attributes can afford different possibilities for action across users and contexts (Dong and Wang, 2018). For example, scholars have classified social media affordances into four categories: visibility, persistence, editability, and relevance (Lin et al., 2013). Based on the new insights into social commerce, Dong and Wang (2018) divided IT affordance into six dimensions, namely visibility, guidance shopping, social connecting, trading, triggered attending, and metavoicing, to explore how these IT features make users achieve their social shopping goals.

Over the recent few years, the affordance term has been widely used in the study of social commerce. Some scholars have studied the relationship building between buyers and sellers from the perspective of IT affordance, and the research results show that the functions of social media greatly affect the formation of strong and weak relationships between buyers and sellers (Shao et al., 2020). Different from traditional e-commerce, social commerce often has a large number of information presentation methods, and the media richness of social commerce can often affect consumers’ viewing intentions (Shao and Pan 2019). In recent years, how to combine the theory of affordances with emerging social business models, such as live streaming shopping, has also attracted much attention of many scholars to explore its underlying causal relationship. For example, Shao et al. (2020) constructed a theoretical model to explore how live streaming influences users’ shopping intention by introducing three specific living streaming features (visibility, metavoicing, and guidance shopping) from the view of IT affordance. Based on the technology provided by the live streaming platform, users can watch the display of goods and interact with sellers, the interaction between buyers and sellers has also become an important aspect affecting consumers’ behavior (Xu et al., 2020). Furthermore, Shao et al. (2020) explored the influence mechanism of user satisfaction and user stickiness in social networks from the perspective of technology affordance and revealed the moderating role of users’ experience in this research model. By providing users with functions that meet their purchase needs, user satisfaction can be greatly improved. On the contrary, the absence of shopping platform functions will reduce users’ participation, which could inhibit the potential for users’ participation. Studies have shown that the IT affordances theory helps explain the technology use effects (Dong and Wang, 2020). The possibility of IT is providing users with a variety of possible purchase goals through specific technology affordances in a certain environment. That is, the more powerful live streaming shopping affordances are, the more active they will stimulate and enable users to engage in social commerce actions. Based on this, combined with the characteristics of live streaming shopping situations, this study explores the impact of visibility, real-time interaction, and guidance shopping on consumers’ purchase intention from the perspective of the IT affordances theory. It also introduces the concept of media richness into the theory of availability; taking live streaming as a social environment, this study explores the impact of affordance in live streaming on consumers’ purchase intention.

### 2.4. Socio-technical interaction perspective

In order to study the influence of technical and environmental characteristics on consumers’ purchasing behavior in live streaming shopping, we introduce a social-technology interaction perspective. Socio-technical interaction perspective holds that the system consists of two parts: social environment and technical characteristics (Wong et al., 2021). Social environment and technical characteristics are often known as mutual support and influence. First of all, from the perspective of technical characteristics, in the process of live streaming shopping, everything about shopping is supported by information technology (Leonardi, 2015). In other words, the live streaming shopping platform is a technology platform (Kong et al., 2019). Specifically, live streaming shopping is carried out with the support of information technology. The development of live streaming shopping business model is inseparable from the support of information technology (Gao et al., 2018). When people do live streaming shopping, the display of the product needs the support of visualization technology, consumer information acquisition needs big data and other technologies to achieve, and user feedback needs comments and other functions to achieve (Cherns, 1976). Second, from the perspective of the social environment, it represents the environment where the system users use and interact. In the context of live streaming shopping, the interaction between users and streamers constitutes the overall social environment. Users can also communicate with each other through bullet screens or comments. In measurement, generally through the degree of interaction, user satisfaction represents the user’s perception of the environment (Chai and Kim, 2012). Some scholars have found that a person’s perception of the social environment can greatly affect the type of behavior, including the adaptation or escape environment (Mehrabian and Russell, 1974). In the process of participating in live streaming, good interaction and communication can encourage users to continue watching. On the contrary, the lack of communication environment is often difficult to establish links between the streamers and users. In the context of live streaming shopping, the influence of the social environment on consumer behavior is significant. The interaction between consumers and the streamer in the process of live streaming shopping, as well as the atmosphere and environment of live streaming, will have an impact on consumers’ purchase behavior (Adelaar et al., 2003). Finally, technology and social environment are interrelated and mutually influenced. In social business activities, technical support provides users with more choices of consumption patterns. The social environment also affects consumers’ purchasing behavior. Technological factors are the antecedent variables that affect consumers to make purchase decisions. Social factors also affect consumers’ behavior. The interaction of the two factors affects consumers’ purchase behavior (Hu and Zhang, 2017). In the context of social commerce, users’ behavior is affected by both social environment and technological characteristics. Most of the existing studies are from a single perspective and lack research on the interaction between technology and social factors. This study is conducted from a socio-technical perspective, choosing guidance shopping, visibility, and media richness to represent the technical factors of live streaming shopping. From the perspective of live streaming technology and social environment interaction, the concept of real-time interaction is introduced. This study hopes to supplement and improve the research on user behavior in live streaming shopping by analyzing the environmental and technical characteristics of live streaming shopping.

## 3. Theoretical model and hypothesis development

### 3.1. Interaction between live streaming technology functions and social situation

Live streaming shopping has changed the way of information exchange in online shopping. The application of live streaming technology combines social media functions with online shopping, and streamers and audiences can exchange information through real-time interaction (Wong et al., 2021). The audience can learn more about the product information. Meanwhile, this kind of chat-like interaction also brings consumers more fun and increases their love for the online shopping mode of live streaming shopping. In this study, we choose real-time interaction as the representative of live streaming technology and social situation interaction.

#### 3.1.1. Real-time interaction, attraction, and cognitive assimilation

Real-time interaction refers to the possibility of timely information exchange between buyers and sellers (Sun et al., 2019). This kind of information exchange can largely eliminate consumers’ uncertainty about live streaming shopping. It can also strengthen the construction of social relations between the two parties. Dong and Wang (2018) believes that interactivity has a significant moderating effect on the formation of strong and weak relationships between buyers and sellers in social purchase activities. Good interaction between buyers and sellers can enhance the strength of the relationship. In the live streaming environment, interaction is an important way of emotional connection between the audience and the streamer. When viewers interact with streamers and other viewers, they have a better impression of the live streaming (Chen and Lin, 2018). Real-time interaction is reflected in the instant communication between consumers and streamers through the barrage and comments. Interaction in social business activities can increase consumers’ perception of the products. Some researchers divide the interaction in social commerce into two types. One type is product reputation and the other is to observe the purchase behavior of other consumers (Lv et al., 2018). Observation of other users’ behaviors enables users to deepen their understanding of the product, and conformity often affects consumers’ purchase choices. Live streaming provides support for these two forms of interaction. Consumers can answer their doubts by asking questions to the streamer. The streamer can also understand customers’ needs through friendly communication and interaction with the audience. Through real-time communication, the streamer can narrow the distance between the audience and provide customers with more shopping guidance (Yim et al., 2017). In the process of live streaming shopping, positive interaction can improve consumers’ interest and recognition of live streaming shopping. Therefore, exploring the impact of real-time interaction on users’ cognition and emotion can help us better understand consumer behavior in live streaming shopping activities. Based on the above viewpoints, we thus propose the following hypotheses:

*H1a*: Real-time interactivity has a positive impact on attraction.

*H1b*: Real-time interactivity has a positive impact on cognitive assimilation.

### 3.2. Live streaming as a technical platform

The mode of live broadcast shopping is realized with certain technical support. First, without the technology of information production and dissemination, such as live streaming, the audience would not be able to interact with the streamers or obtain product information through live streaming. Second, the streamers provide opinions on product selection through live streaming. The audience raises their questions and purchase demands through bullet screens, and the streamers provide answers and professional opinions through live streaming. Finally, live streaming shopping incorporates text, pictures, voice, video, and other social media information. Live streaming shopping not only realizes the function of online shopping but also realizes the function of social media. This study chooses visibility, guidance shopping, and media richness as the technical characteristics of live streaming shopping.

#### 3.2.1. Guidance shopping and attraction, cognitive assimilation

Guidance shopping refers to the possibility of providing consumers with appropriate product choices or services based on their needs and preferences (Dong and Wang, 2018). In live streaming shopping, streamers can provide customers with personalized recommendations based on their actual needs and personal preferences, which helps to enhance the customer’s cognitive experience and allows customers to immerse themselves in the consumption situation (Wang and Yu, 2017). Guidance shopping in live streaming shopping is mainly achieved through two ways: one is to provide product and service recommendations according to customers’ consumption habits. Recommendation information provided in this way often lags behind in time and is increasingly unable to meet consumers’ shopping needs. The other way is to provide consumers with shopping choices based on demand and interest through the introduction. The streamer understands the needs of consumers through real-time interaction with consumers. Guidance to consumers can increase consumers’ perception of product information by recommending appropriate products to consumers, thereby increasing the social existence of consumers in live streaming shopping (Ou et al., 2014). By providing customers with shopping guidance to enhance consumers’ stickiness in using live streaming shopping, consumers will be attracted by the product information in which they are interested. Guidance shopping is a comprehensive display of information exchange in live streaming shopping, which meets consumers’ shopping needs through guidance shopping. Based on the above viewpoints, we thus propose the following hypotheses:

*H2a*: Guidance shopping has a positive impact on attraction.

*H2b*: Guidance shopping has a positive impact on cognitive assimilation.

#### 3.2.2. Visibility and attraction, cognitive assimilation

Visibility is defined as the degree of information display about products, including text, pictures, videos, and other information visible to consumers (Dong and Wang, 2018). Consumers can get timely updated product information when they are in live streaming shopping. Consumers get information about products visually. Live streaming shopping is an all-round display of products based on the visualization of product information. Consumers can understand the use of products and see the use of the effect of products according to the presentation of the streamer. Visual information stimulation greatly reduces the uncertainty of consumers on products (Yim et al., 2017). Live streaming shopping focuses on providing consumers with a sense of reality in the product information display. In live streaming, the consumer can see the streamer throughout the whole process. As a real individual, the streamer enhances the social existence perceived by the consumer (Li, 2019). In the live streaming shopping process, consumers get a full display of product information, the two-dimensional information became a three-dimensional perception of the product (Sun et al., 2019). Based on the above viewpoints, we thus propose the following hypotheses:

*H3a*: Visibility has a positive impact on attraction.

*H3b*: Visibility has a positive impact on cognitive assimilation.

#### 3.2.3. Media richness and attraction, cognitive assimilation

The concept of media richness originates from the theory of information richness theory, which is defined as the ability of information to change users’ understanding and communication through media communication (Daft and Lengel, 1986). Information is transmitted through media to provide users with perceptual cues. For example, in the live streaming shopping situation, consumers can get information through text, pictures, videos, live screens, and other ways to enrich their cognition. From the perspective of information dissemination, different media have different impacts on the dissemination ability of information. It is generally believed that face-to-face information dissemination is the most effective means of information dissemination because it allows rapid mutual feedback (Song et al., 2004). Shao et al. (2019) believes that in a social media interaction, social media richness will affect users’ access to information and perception. Specifically, the media richness provides users with a richer social media experience, which will affect users’ participation behavior. From the perspective of network media and information technology, the continuous updating of network media and the rapid development of information technology provides the possibility for multi-form media communication. The higher degree of media richness, the more information perception and media experience can be given to users. On the contrary, the low degree of media richness may not only produce the rapid dissemination of information but also affect the media experience of users (Tseng and Wei, 2020). Through the diversified media dissemination of the live streaming platform, users are given rich information perception from various aspects such as vision and hearing. Diversified information presentation will increase consumers’ experience, reduce users’ uncertainty due to insufficient information acquisition, and improve the attractiveness of rich media communication paths to consumers. According to the theory of media richness, people tend to choose media with higher media richness, which will also lead to more active social interaction and other participation behaviors (Anandarajan et al., 2010). Based on the above viewpoints, we thus propose the following hypotheses:

*H4a*: Media richness has a positive impact on attraction.

*H4b*: Media richness has a positive impact on cognitive assimilation.

### 3.3. Attraction, cognitive assimilation, and purchase intention

In the application of the socio-technical perspective, users’ cognition, emotion, and behavior are the main research directions. Users’ emotional tendencies and cognitive attitudes are the two main aspects affecting users’ behavior (Leonardi, 2015). Users’ perception of technology and social environment affects their own emotions and cognition, which is ultimately reflected in the guidance of users’ behavior. In the live streaming shopping scene, attraction represents that the audience is willing to continue to watch the live streaming. Attraction can predict people’s future attitudes and behaviors while making people have emotional tendencies (Shen et al., 2019). As an expression of emotional tendency, the attraction has been widely used in existing studies to represent emotional tendency (Wang and Han, 2021). In this study, attractiveness is taken as a variable representing consumers’ emotional tendencies, which reflect the important influence of the attractiveness of live streaming shopping on consumers’ purchasing behavior. Cognitive assimilation is used as the expression of cognitive attitude (Wang and Yu, 2017). Emotional tendency represents the user’s emotional response; cognitive attitude is the embodiment of the user’s self-perception (Adelaar et al., 2003). In live streaming shopping, cognitive assimilation represents the consumers’ knowledge and understanding of products, which is another aspect that affects consumers’ purchasing decisions. Therefore, this study chooses cognitive assimilation to represent consumers’ cognitive attitudes. Based on the S-O-R model, stimulus to response is affected by the body state. This study argues that attraction and cognitive assimilation as internal organic states stimulate the generation of consumers’ purchase intention.

Some scholars defined consumers’ activities from three aspects: cognition, emotion, and behavior (Lv et al., 2018). Consumers access product information through a variety of media (Adelaar et al., 2003). In the context of live streaming shopping, consumers can access product and service information through text descriptions, pictures, and live streaming by streamers (Xu et al., 2020). In the context of live streaming shopping, consumers’ purchase decisions are affected by technical factors and social factors. For example, when the audience is attracted by a live advertisement (text, picture) to start a live streaming shopping, the live audience becomes a potential consumer. After watching the live streaming, the live audience may purchase the live commodity for reasons such as actual demand or impulse consumption. In the whole process, the audience’s subjective cognition will be affected by various media information, streamers, or other comments. At the same time, emotional tendencies affect consumers’ subjective perception of goods, and positive emotional tendencies can make consumers more willing to accept product recommendations. Based on the above viewpoints, we thus propose the following hypotheses:

*H5*: Attraction has a positive impact on purchase intention.

*H6*: Cognitive assimilation has a positive impact on purchase intention.

### 3.4. Mediating effect of attraction and cognitive assimilation

Previous studies have found that technology and social factors have an impact on consumers’ purchase intention, and this impact is realized by attraction and cognitive assimilation (Xu et al., 2020). Attraction is the most critical factor to encourage consumers to buy. Attracting consumers’ interest and curiosity in products through various ways is the basis to guide consumers to further understand products (Adelaar et al., 2003). Consumers’ cognition of products is also a way to influence consumers’ purchase intention. Cognitive assimilation changes consumers’ attitudes toward products by influencing their perceptions of products (Lv et al., 2018).

In the context of live streaming shopping, the influence of technological factors and social factors on consumers’ purchasing behavior is usually realized through attraction and cognitive assimilation (Xu et al., 2020). Attractiveness is the starting point of establishing the connection between buyers and sellers. In the process of live streaming shopping, the streamer attracts consumers’ attention through technological and social factors and thus increasing the possibility of purchase. The process of users watching live streaming shopping is more about the formation of their cognition of the product. Through the introduction of streamers in live streaming, consumers will have a more profound and comprehensive cognition of the product. Influencing consumers’ cognition of products through technological means and social interaction is also a way to influence consumers’ purchase intention. Based on the above viewpoints, we thus propose the following hypotheses:

*H7*: Attraction mediates the relationship between real-time interaction, guidance shopping, visibility, media richness, and purchase intention.

*H8*: Cognitive assimilation mediates the relationship between real-time interaction, guidance shopping, visibility, media richness, and purchase intention.

In order to test the structural model and reduce the influence of non-study variables on the data analysis, our study included eight control variables. The control variables were gender, age, education background, monthly income, whether they knew about live streaming shopping, the time they used live streaming shopping, the number of times they used live streaming shopping, and the live streaming software they used.

**Figure 1:**
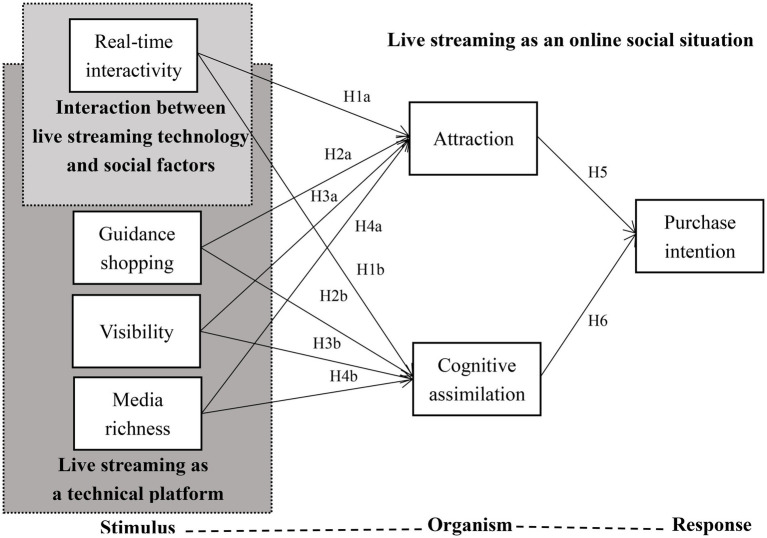
Research model.

In the context of live streaming shopping, technical factors have an impact on live streaming shopping behavior through information technology. Based on the stimulus-organism-response (S-O-R) framework, from the perspective of socio-technology interaction, we construct a theoretical model from three levels: live streaming as a technical platform, the interaction between live streaming technical function and social context, and live streaming as an online social environment. The technical factors mainly include visibility, guidance shopping, and media richness, and the social-technology interaction factors mainly include real-time interaction. The user’s perceived attraction and cognitive assimilation are the internal organism state, and the purchase intention of live streaming is the response. Figure 1. shows the relationship between the variables involved in this study.

## 4. Research methodology and date analysis

### 4.1. Research method

This study mainly adopts a survey for data collection. In order to ensure the content validity and measurement validity of constructs, the measurement items of this study are all adapted from the existing literature. By combining the situation of live streaming shopping, we designed a questionnaire that fits our study. In this study, we use a seven-point Likert Scale to measure the focus constructs. This study involved seven constructs. The measurement items of real-time interaction (RI), visibility (VI), purchase intention (PI), and guidance shopping (GS) were adapted from Dong and Wang (2018). The item of media richness (MR) was adapted from Shao and Pan (2019). Attraction (AT) and Cognitive assimilation (CA) were measured by items adapted from Xu et al. (2020). In order to get valid questionnaires, our study designed reverse items to ensure the effectiveness of the questionnaire. In order to ensure the accuracy of the questionnaire items, we did a pre-test experiment. We randomly invited 20 survey subjects who had participated in live streaming shopping for a test and modified the scale according to the test results to ensure the accuracy of the questionnaire scale. The measurement items involved in the questionnaire scale are shown in Appendix A.

When collecting survey data, we published the questionnaire on China’s largest questionnaire platform “Sojump” and spread the link through the social platforms WeChat and QQ. We prepared random lucky money for the respondents to encourage them to engage. A total of 425 questionnaires were collected from 9 April 2021 to 25 May 2021. In addition, we set the screening question “Have you ever had a live streaming shopping experience” to screen the survey subjects. A total number of 223 valid questionnaires were obtained after excluding invalid questionnaires. The descriptive statistics of the sample are shown in Table 2. It can be seen from Table 2 that most of the respondents are female users (68.6%). Most respondents were in their 20 and 30 s. Most of them have known about live streaming shopping (88.8%). The commonly used platforms for live streaming shopping are Tao bao live streaming (68.6%) and Dou Yin live streaming (54.3%). Characteristics of users are consistent with the research situation of live streaming shopping.

**Table 2:** Sample characteristics description.

Demographic variable	Category	Frequency	Percentage (%)
Sexuality	Man	70	31.4%
	Women	153	68.6%
Age	<20	47	21.2%
	20–30	131	58.7%
	30–40	33	14.8%
	40–50	11	4.9%
	>50	1	0.4%
Education	Senior high school	17	7.6%
	Junior college	19	8.5%
	Bachelor	145	65.0%
	Masters	33	14.8%
	PhD	9	4.0%
Live streaming shopping experience	3 months or less	81	36.3%
	4–6 months	35	15.7%
	7–12 months	15	6.7%
	1–2 years	68	30.5%
	2 years or more	24	10.8%
Monthly income (¥)	<2,000	107	48.0%
	2,000–5,000	53	23.8%
	5,000–8,000	33	14.8%
	>8,000	30	13.5%
Number of live streaming shopping per month	0	72	32.3%
	1–3	82	36.8%
	3–5	24	10.8%
	5–7	29	13.0%
	>7	16	7.2%
Used live streaming shopping software	Taobao	153	68.6%
	WeChat	30	13.5%
	Tik Tok	121	54.3%
	Kwai	60	26.9%
	Others	37	16.6%

### 4.2. Data analysis method

The selection of data analysis methods has an important impact on the reliability of research results. Because the measurement items used in this study are mostly from the existing literature. Due to the different research situations, it is necessary to ensure the effectiveness of the measurement items used in the live streaming situation concerned in our study. Therefore, this study first used SPSS25.0 software for exploratory factor analysis, deleted invalid load factor items (including error factor load and double factor load), and finally got the formal scale used in this study (see Appendix A; Chin, 1998). In the part of the structural model test, our study uses Amos25.0 software for confirmatory factor analysis. Through confirmatory factor analysis, the reliability and effectiveness of the structural model and the path coefficient between relevant factor variables can be effectively tested (Gefen et al., 2000a,b).

#### 4.2.1. Measurement model

In order to ensure that the measurement model can properly reflect the observed variables in this study, exploratory factor analysis was used to evaluate the validity and reliability. The main parameters include Cronbach’s α and composite reliability (CR; Bagozzi and Yi, 1988). The values of Cronbach’s α and CR of all variables should be >0.7, which indicates that the construct has good reliability. All the variables measured in our study are greater than the threshold of 0.7. This shows that our research data have good reliability. The results are shown in Table 3.

**Table 3:** Reliability and validity analysis of construct.

Construct	Indicator	Substantive factor loading	Cronbach’s *α*	CR	AVE
Real-time interaction	RI1	0.554	0.785	0.784	0.500
	RI2	0.739			
	RI3	0.718			
	RI4	0.739			
Guidance shopping	GS1	0.684	0.816	0.828	0.547
	GS2	0.738			
	GS3	0.762			
	GS4	0.772			
Visibility	VI1	0.729	0.795	0.815	0.527
	VI2	0.786			
	VI3	0.790			
	VI4	0.580			
Media richness	MR1	0.710	0.711	0.742	0.500
	MR2	0.781			
	MR3	0.603			
Attraction	AT1	0.683	0.809	0.805	0.514
	AT2	0.775			
	AT3	0.830			
	AT4	0.548			
Cognitive assimilation	CA1	0.724	0.800	0.807	0.514
	CA2	0.775			
	CA3	0.780			
	CA4	0.568			
Purchase intention	PI1	0.677	0.824	0.817	0.599
	PI2	0.843			
	PI3	0.793			

The convergent validity of variables is evaluated by testing factor loading and average variance extracted (AVE; Chin et al., 2003). The factor loading values of all variables were higher than 0.7, and all AVE values were higher than 0.5, which indicates that the measurement model had good aggregation validity. The factor loading values of all variables involved in the measurement model were higher than 0.5, and the AVE values were about 0.5. In the evaluation of the discriminant validity of each variable in the measurement model, our study uses the cross-load coefficient of the measurement index Fornell Larcker criterion to compare and analyze whether the square root of each variable AVE is greater than the correlation coefficient between the variable and other variables (Hair et al., 2014). Through testing, the factor-load coefficients of all the measurement indicators used in our study and their corresponding variables are significantly higher than those of the indicators and other variables. At the same time, the square root of the AVE value of each variable is greater than the correlation coefficient between the variable and other variables. The results are shown in Tables 4, 5.

**Table 4:** Cross-factor loading analysis.

Construct	RI	GS	VI	MR	AT	CA	PI
RI1	0.751	0.423	0.271	0.247	0.372	0.195	0.366
RI2	0.802	0.371	0.401	0.372	0.362	0.321	0.349
RI3	0.806	0.450	0.394	0.410	0.282	0.226	0.272
RI4	0.767	0.444	0.298	0.288	0.275	0.218	0.251
GS1	0.476	0.794	0.203	0.328	0.282	0.222	0.234
GS2	0.429	0.769	0.330	0.373	0.267	0.334	0.300
GS3	0.381	0.806	0.307	0.356	0.240	0.340	0.288
GS4	0.449	0.851	0.346	0.418	0.308	0.297	0.291
VI1	0.272	0.258	0.782	0.364	0.267	0.383	0.213
VI2	0.332	0.313	0.823	0.405	0.340	0.317	0.281
VI3	0.342	0.220	0.815	0.307	0.225	0.264	0.218
VI4	0.424	0.351	0.733	0.278	0.247	0.306	0.329
MR1	0.378	0.391	0.453	0.832	0.319	0.367	0.320
MR2	0.334	0.353	0.310	0.842	0.287	0.215	0.333
MR3	0.285	0.350	0.262	0.710	0.351	0.373	0.224
AT1	0.314	0.182	0.266	0.283	0.763	0.390	0.381
AT2	0.367	0.327	0.305	0.402	0.832	0.365	0.393
AT3	0.319	0.313	0.280	0.319	0.867	0.420	0.343
AT4	0.325	0.261	0.240	0.249	0.725	0.387	0.375
CA1	0.264	0.299	0.348	0.362	0.458	0.812	0.277
CA2	0.131	0.241	0.268	0.280	0.351	0.806	0.211
CA3	0.240	0.229	0.301	0.266	0.357	0.802	0.247
CA4	0.342	0.386	0.364	0.326	0.382	0.742	0.306
PI1	0.394	0.322	0.362	0.337	0.437	0.362	0.828
PI2	0.325	0.261	0.308	0.293	0.379	0.237	0.898
PI3	0.323	0.309	0.194	0.336	0.402	0.264	0.857

RI, Real-time interaction; GS, Guidance shopping; VI, Visibility; MR, Media richness; AT, Attraction; CA, Cognitive assimilation; PI, Purchase intention.

**Table 5:** Correlation coefficients between variables.

	RI	GS	VI	MR	AT	CA	PI
RI	0.692						
GS	0.540	0.740					
VI	0.435	0.363	0.726				
MR	0.418	0.456	0.430	0.702			
AT	0.416	0.341	0.342	0.395	0.717		
CA	0.307	0.365	0.404	0.390	0.489	0.717	
PI	0.399	0.342	0.331	0.371	0.168	0.328	0.774

Diagonal bold value is the square root of the AVE value of each variable.

#### 4.2.2. Common method bias

Common method bias refers to the artificial covariation between predictor and outcome variables caused by the same data source or raters, the same measurement environment, the context of the survey variable, and the variable itself (Podsakoff et al., 2003). This study reduces the generation of common method bias from two aspects: program control and statistical test. First, from the aspect of program control, at the beginning of the questionnaire design, it is necessary to consider reducing the common method deviation. In the process of a questionnaire survey, anonymous evaluation is adopted. The length of the problem and the order of the problem are set reasonably. Second, our study tests the common method bias in the measurement model from the perspective of statistics. Our study first uses the Harman single factor test method to evaluate the influence of common method bias by calculating the maximum variance interpretation rate of a single factor in the model (Podsakoff et al., 2003). The results show that the maximum variance interpretation rate of the single factor in the model is 31.33%, which does not account for the majority of the total variance. Therefore, the common method bias has no significant influence on the measurement model of this study. Finally, this study examines whether there is a common method bias in the model by confirmatory factor analysis (Wen and Dandan, 2018). A latent variable with common method deviation was added to the structural equation model, and the adaptation index of the original model and the structural model with the latent variable was compared. The test results showed that the change of RMSEA did not exceed 0.05, and the change of CFI and IFI did not exceed 0.1. The results are shown in Table 6, indicating that there was no obvious common method deviation in the measurement model of this study.

**Table 6:** Common method deviation test.

	GFI	AGFI	RMSEA	IFI	CFI
Original model	0.863	0.831	0.055	0.919	0.917
Model after adding common method factor	0.891	0.850	0.045	0.951	0.949

#### 4.2.3. Structural model

**Figure 2:**
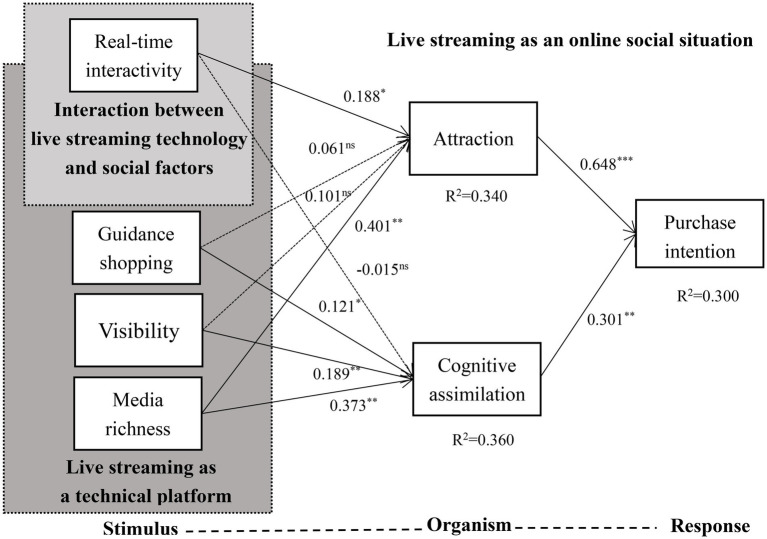
Model test results. ***p < 0.001; **p < 0.05; *p < 0.1; ns *p* > 0.1; Solid arrows represent significant influence; dotted arrows represent insignificant influence.

In our study, Amos25.0 software was used to analyze the structural model. The data were analyzed to test the significance of the relationship between measurement variables and the fitting index of the model. The specific analysis results are shown in Table 7. According to the fitness index of the model, it can be seen that the value of the variable correlation index used in our study is within the recommended value range, so the model in this study has a good fitting degree. The explicitness of the relationship between variables is shown in Figure 2. *R*^2^ represents the extent to which structural models account for variations in variables (Gefen et al., 2000a). It can be seen from the values in the figure that the *R*^2^ value of the entire model is 0.3, indicating that this model explains 30% of the variation in consumers’ purchase intention. The *R*^2^ value of attraction is 0.34, and the *R*^2^ value of cognitive assimilation is 0.36, indicating that the four variables of visibility, real-time interaction, guidance shopping, and media richness have strong explanatory power for attraction and cognitive assimilation.

**Table 7:** Structural model adaptation index.

Adaptation indicators	Recommended values	Fitting values
*λ* ^2^	The smaller the better	474.883
*λ*^2^/df	<3.000	1.678
GFI	>0.800	0.863
AGFI	>0.800	0.835
RMSEA	<0.080	0.055
NNFI	>0.800	0.820
IFI	>0.900	0.919
CFI	>0.900	0.917

From the above analysis, it can be seen that the path coefficient of real-time interaction on attraction is 0.188 (*p* < 0.1), indicating that real-time interaction has a positive impact on attraction. The hypothesis of H1a is supported. The path influence coefficient of real-time interaction on cognitive assimilation is −0.015 (*p* > 0.1), indicating that the influence of real-time interaction on cognitive assimilation is not significant, and that hypothesis of H1b is not supported. The path coefficients of guidance shopping on attraction and cognitive assimilation were 0.061 (*p* > 0.1) and 0.121 (*p* < 0.05), respectively. The results indicate that guidance shopping had no significant effect on attraction, and the conclusion did not support the hypothesis of H2a. It also shows that guidance shopping has a significant effect on cognitive assimilation, which supports the hypothesis of H2b. The effect of visibility on attraction was not significant (*β* = 0.101, *p* > 0.1). Visibility had a significant effect on cognitive assimilation (*β* = 0.189, *p* < 0.05). It can be seen that visibility has no positive effect on attraction, hypothesis H3a is not supported. Visibility has a positive effect on cognitive assimilation, hypothesis H3b is supported. The path influence coefficients of media richness on attractiveness and cognitive assimilation are 0.401 (*p* < 0.05) and 0.373 (*p* < 0.05), indicating that media richness has a significantly positive impact on the attractiveness and cognitive assimilation. Hypotheses H4a and H4b are supported.

In the relationship between attraction and consumers’ purchase intention, the path coefficient of attraction to consumers’ purchase intention is 0.648 (*p* < 0.001), which indicates that attraction has a significant positive impact on consumers’ purchase intention, and hypothesis H5 is supported. The path coefficient of cognitive assimilation on consumers’ purchase intention was 0.301 (*p* < 0.05), which indicated that cognitive assimilation had a positive effect on consumers’ purchase intention, hypothesis H6 is supported. The influence of attraction and cognitive assimilation on purchase intention is significant, and the results of statistical analysis are shown in Table 8. By comparing the effects of attraction and cognitive assimilation on purchase intention, we found that attraction has a more significant impact on consumers’ purchase intention.

**Table 8:** Significance test of mediating variable to the dependent variable.

	PI		
	*β*	*t*	Value of *p*
AF	0.520	5.958***	0.000
CA	0.186	1.932***	0.000
F		33.183***	

AT, Attraction; CA, Cognitive assimilation; PI, Purchase intention.*** means the *t*-value is significant.

In this study, process plug-in in SPSS was used to test the mediating effect of the structural equation model within a 95% confidence interval as shown in Table 9.

**Table 9:** Mediating effect test results.

	Effect size	Boot SE	Boot LLcl	Boot ULcl
RI → AF → PI	0.198	0.047	0.120	0.307
RI → CA → PI	0.091	0.046	0.023	0.208
GS → AF → PI	0.172	0.042	0.102	0.266
GS → CA → PI	0.109	0.049	0.035	0.228
VI → AF → PI	0.175	0.045	0.097	0.270
VI → CA → PI	0.120	0.048	0.040	0.237
MR → AF → PI	0.201	0.049	0.120	0.308
MR → CA → PI	0.113	0.046	0.034	0.217

The indirect effects of real-time interaction, guidance shopping, visibility, and media richness on consumers’ purchase intention through attraction and cognitive assimilation are not included in the 95% confidence interval, which indicates that there is a significant mediating effect in the structural model. Hypotheses H7 and H8 are supported.

In order to test whether attraction and cognitive assimilation play a partial mediation effect or a complete mediation effect, our study uses Baron and Kenny (1986) method to test the relationship between independent variables and dependent variables in the presence of intermediary variables. First, by examining the relationship between independent variables and mediating variables, we found that real-time interaction, guidance shopping, visibility, and media richness have significant effects on attraction and cognitive assimilation. Second, when there is no intermediary variable, the effect value of the independent variable on the intermediary variable, the effect between the real-time interaction, guidance shopping, visibility, media richness, and purchase intention are significant. Finally, with the addition of the mediating variables, attraction and cognitive assimilation, the effect values of real-time interaction, guidance shopping, visibility, and media richness on purchase intention are significantly reduced, indicating that attraction and cognitive assimilation play a partial mediating role between real-time interaction, guidance shopping, visibility, media richness, and consumers’ purchase intention. The test results are shown in Table 10.

**Table 10:** Partial mediation effect and complete mediation effect test results.

	AF effect	CA effect	PI (no mediation) effect	PI (mediation) effect	*t*
RI	0.421	0.282	0.307	0.214	3.691***
GS	0.336	0.326	0.239	0.195	0.959**
VI	0.338	0.363	0.256	0.202	2.658**
MR	0.411	0.369	0.271	0.226	3.111**

*** means the *t*-value is significant.

## 5. Discussion

### 5.1. Research conclusions

From the perspective of socio-technical, this study establishes a theoretical model and conducts empirical tests to explore the influence of social and technological factors on consumers’ purchase intention in live streaming shopping. Through testing the research hypothesis, we got a relevant conclusion.

First, real-time interaction has a positive impact on attractiveness but has no significant impact on cognitive assimilation. Real-time interaction is an effective way to establish an emotional connection between buyers and sellers. At the same time, real-time interaction can strength the relationship between buyers and sellers. In the live streaming situation, the good interaction between the streamer and the audience is an effective way to enhance the attraction of live streaming shopping to consumers. The streamer reduces the consumers’ strangeness through self-introduction and product display; it can also close the psychological distance between them. The social connection between buyers and sellers through real-time interaction is a kind of emotional connection between the two sides, which is more stable and reliable than an ordinary business relationship. This study analyzes the particularity of live streaming shopping mode. Our research finds that the product information presented in the form of live streaming can attract consumers’ attention. In live streaming shopping, the impact of real-time interaction on cognitive assimilation is not significant. We believed that there are several possible reasons for this. First, in live streaming shopping, consumers will interact with other viewers while interacting with streamers, and information from different sources may produce conflict; the amount of information available in a short period of time can create cognitive difficulties for consumers, which will confuse consumers’ cognition (Xu et al., 2020). Second, in live streaming shopping, streamers often introduce multiple products in a short time, and the product information provided by streamers varies for different goods. When the product information introduced by streamers to consumers is not comprehensive enough and the streamers fail to solve the wonders of consumers through interaction, it is not easy for consumers to have cognitive assimilation, but it needs time.

Second, guidance shopping has a positive effect on cognitive assimilation but has no significant effect on attractiveness. Guidance shopping is an important factor in the theory of IT affordance. From the perspective of IT affordance, guidance shopping is to provide consumers with personalized product recommendations according to consumers’ needs and preferences for products. The two sides solve the problems generated by consumers in the shopping process through further exchanges, so as to better meet their needs. In the process of providing shopping guidance to consumers, the relationship between streamer and audience is more closely. From the perspective of products, the reason why consumers choose live streaming shopping is largely due to their own needs. Before making shopping choices, consumers often have a subjective judgment on whether the product is suitable for them. The director’s shopping guidance only makes consumers clear about their shopping choices, which explains the reason why guidance shopping has no obvious influence on attraction.

Third, visibility has a significant positive impact on cognitive assimilation but has no significant impact on attraction. Visibility is one of the relevant technical characteristics in the field of social commerce. From the perspective of social commerce, visual perception is the most direct way for consumers to obtain goods or service information. Consumers produce direct impressions of the product through the information they see. The product information provided by visibility for consumers can effectively reduce consumers’ perceived risk and uncertainty of products (Dong and Wang, 2018). In the live streaming environment, consumers can obtain a lot of product information through live streaming. For example, consumers can only see the effect of products through pictures and videos before. However, in live streaming shopping, the streamer can provide consumers with a live display of product effects according to the specific situation of consumers. Consumers will process and organize the contents they see so as to deepen their own cognition or correct their previous perception. Compared with the positive effect of visibility on cognitive assimilation, the impact of visibility on attractiveness is not significant in the live streaming shopping context (Sun et al. 2019). In the previous online shopping process, consumers can only understand the product information through pictures, text introductions, and other ways. Whether it is from the perspective of the seller or the perspective of the buyer, due to the limitations of information dissemination, the content and volume of information are very limited, and consumers can quickly complete the acquisition and processing of commodity information. In the network of live streaming, due to the change in the way of information dissemination, the main content to the audience is often the display of dynamic effects. The audience needs to spend a certain amount of time and energy to collect information about commodities, which undoubtedly increases the difficulty of consumers in obtaining commodity information, resulting in the dispersion of consumer attention. It is difficult to attract consumers’ attention the first time.

Fourth, media richness has a significantly positive impact on attractiveness and cognitive assimilation. In the live streaming situation, both businesses and consumers have rich media information expression. The streamer can introduce product information through text, pictures, and live streaming videos. The audience can also express their ideas through rich media (Shao et al., 2020). Media richness is a technical feature that online live streaming shopping is more characteristic of other e-commerce models. Live streaming integrates all media information expressions, providing consumers with richer sources of information and facilitating communication between streamers and customers. Diversified media information expression can satisfy consumers’ curiosity, eliminate uncertainty, and increase the attraction of live streaming shopping to consumers. More information acquisition means that consumers can form their own cognition of products more quickly and comprehensively, which also helps to increase consumers’ understanding of products.

Fifth, attraction and cognitive assimilation have a positive impact on consumers’ purchase intention. Attraction is the highlight of live streaming shopping compared with other social business models. Due to its more entertaining nature, live streaming shopping meets consumers’ shopping needs and their own entertainment needs, which will bring more satisfaction to consumers. Meanwhile, the emotional connection based on interaction is often more solid than the single communication based on text, which can provide consumers with a sense of social existence. Online live streaming eliminates consumers’ uncertainty in the shopping process through more abundant product information displays. It also urges consumers’ cognition of products more comprehensive. This cognition is produced after consumers watch live streaming shopping. In terms of time, it is subjective cognition that can most affect consumers’ purchase intention. The survey results show that consumers’ own cognitive assimilation is an important prerequisite for influencing consumers’ behavior. Cognitive assimilation based on information acquisition will affect consumers’ subsequent social sharing and have an important impact on their willingness to consume.

Finally, attraction and cognitive assimilation mediate the relationship between visibility, media richness, guidance shopping, real-time interaction, and consumer purchase intention. Attraction and cognitive assimilation can affect the impact of social and technical factors on consumer purchase intention. Technical factors and social factors in live streaming shopping are the direct factors affecting consumers’ purchase intention, which are realized by attraction and cognitive assimilation. Live streaming shopping mode attracts the attention of consumers with its own characteristics. Consumers form their own cognition through the acquisition of live streaming shopping information. This cognition is largely affected by technical factors and social factors in live streaming shopping mode. Attraction and cognitive assimilation change the influence of social and technical factors on consumers’ purchase intention to some extent.

### 5.2. Theoretical implications

The theoretical contributions of this research mainly focus on the following aspects.

First, from the perspective of socio-technical interaction, this study discusses the factors that affect consumers’ purchase intention in live streaming shopping. Although there have been studies from a technical point of view to explore the technical characteristics of live streaming shopping on the impact of consumer purchase intention, there has been a lack of research on the social characteristics of live streaming shopping (Li et al., 2021). Most of the research focuses on social characteristics the impact of trust, and perceived risk on consumer purchase behavior (Xu et al., 2020). There is a lack of theoretical integration between technical and social perspectives. Based on the practice of live streaming situations, this study explores the interactive logic of socio-technical interaction from three levels: live streaming as a technical platform, the interaction between live streaming technical functions and social situations, and live streaming as an online social situation. It further details and reveals the impact of social and technical factors on purchase decisions in the process of live streaming shopping. The technical factors and social factors of live streaming shopping are equally important. They influence and support each other and together constitute the environment of live streaming shopping. The discussion on the application of the socio-technical perspective in this study enriches the related research on consumer behavior, providing a theoretical reference for the subsequent research on consumer purchase behavior in live streaming shopping.

Second, this study introduces consumer emotion into the study of consumer behavior and confirms that the emotional attitude of consumers in the process of live streaming shopping will greatly affect their final purchase decision. Although there have been studies on the factors affecting consumers’ behavior from many aspects, there is a lack of discussion on the influence of emotional tendencies on consumer decision-making (Kong et al., 2019). In the new e-commerce format, consumers are increasingly inclined to choose the emotional connection established by the buyers and sellers as the basis for their shopping choices. In the live streaming situation, the emotional connection between the streamer and the audience will directly affect the emotional tendency of consumers.

Third, this study expands the theoretical application of technology affordance theory in live streaming shopping situations. Although previous studies have researched the impact of IT affordance theory on consumer purchase behavior, with the support of information network technology, the new social commerce model has solved many problems that cannot be solved before (Sun et al. 2019). In the scene of live streaming shopping, the introduction of IT affordance theory can well explain the influence of technology factors on consumer behavior. Meanwhile, our study also introduces the concept of media richness into the IT affordance theory, reflecting the rich application of information technology means in the live streaming scene, and enriches the research of IT affordance theory in the scene of live streaming shopping.

At the same time, based on the theory of IT affordance, this study also discusses the interaction between technical functions and social emotions in the live streaming scene and further refines the theoretical research on live streaming shopping.

### 5.3. Practical implications

Through the discussion of the research results, the practical implications of this study mainly concentrate on the following aspects. First, this study helps enterprises to understand the relationship between social and technical characteristics in live streaming shopping scenes. For example, in terms of consumer perception, the richer product information is expressed, the more consumer uncertainty is eliminated (Gefen and Straub, 2004). When displaying commodities, the live streaming shopping platform should fully display commodity information as much as possible, which includes various attributes of commodities, use effect, and related after-sales service. Only sufficient details can meet consumers’ demand for product understanding. Live streaming shopping is a hot spot in current mobile social commerce. In order to attract consumers’ attention, interesting things must be displayed in the live scene. How to fully display the production to consumers is a little inspiration that this study wants to bring to relevant enterprises.

Second, the research results help enterprises to better apply live streaming technology and expand the channels of product promotion. The great enrichment of material living standards has changed people’s purely demand-oriented shopping style. In social business activities, people pay more attention to the experience in specific scenarios. Enterprises involved in online shopping should shift their focus from “traffic” to “context” to meet the needs of consumers and create a more diversified live streaming shopping scene. As one of the marketing scenarios, live streaming can quickly attract consumers’ attention. How to create content product information around consumers’ psychology, fully connect with consumers’ values and create live streaming content according to consumers’ value needs is very important for product marketing of enterprises.

### 5.4. Limitations and future research

The research of this study has the following limitations: First, the respondents in this study are people with the live streaming shopping experience and there is a lack of surveys on users who use other online shopping models. Whether the survey conclusion is applicable to consumers of other new online business models needs further demonstration. Second, due to the complexity of the live streaming shopping environment and the diversity of participants, consumers’ purchase intention cannot completely represent their final purchase behavior. Therefore, longitudinal studies on consumer behavior at different time periods can be added to future research, in order to have a clearer understanding of consumer behavior. Finally, streamers are also important factors affecting consumers’ purchasing behaviors in live streaming shopping. Their professional and trustworthy characteristics may affect consumers’ final purchasing decisions. Future studies can start with various subjects participating in live streaming shopping to study the influence of the characteristics of different subjects on consumers’ purchasing behaviors.

## 6. Conclusion

From the perspective of socio-technical interaction, this study explores the technical and social factors that affect consumers’ purchase intention in live streaming shopping scenes. Through the study, we found that technical factors and social factors do not affect consumer purchase intention alone. Under the interaction of technical and social factors, the characteristic factors in live streaming shopping will affect consumers’ shopping choices. This study not only expands the theoretical research on consumer behavior in the context of live streaming shopping but also provides practical guidance for enterprises to better apply live streaming technology for product marketing.

## Data availability statement

The raw data supporting the conclusions of this article will be made available by the authors, without undue reservation.

## Author contributions

XD: conceptualization, theoretical foundation, and investigation. XL: methodology and writing. XX: writing. All authors contributed to the article and approved the submitted version.

## Funding

This work was supported by the grants from the National Natural Science Foundation of China 71902158, 72272122, 71972061, and 72172043. This work was supported in part by the Guangdong Basic and Applied Basic Research Foundation under grant 2019A1515110992.

## Conflict of interest

The authors declare that the research was conducted in the absence of any commercial or financial relationships that could be construed as a potential conflict of interest.

## Publisher’s note

All claims expressed in this article are solely those of the authors and do not necessarily represent those of their affiliated organizations, or those of the publisher, the editors and the reviewers. Any product that may be evaluated in this article, or claim that may be made by its manufacturer, is not guaranteed or endorsed by the publisher.

## Appendix A

Constructs and measurement items.

**Table:**

Real-time interaction (Dong and Wang, 2018)	1. During live streaming shopping, I can communicate well with streamers
	2. During live streaming shopping, when I communicate with streamers, I can choose to watch the information of the products I am interested in
	3. During live streaming shopping, streamers facilitate two-way communication between me and him/her
	4. During live streaming shopping, the streamer allows me to question his or her remarks
Guidance shopping (Dong and Wang, 2018)	5. During live streaming shopping, streamers can provide me with information about other alternatives to the product I want to buy
	6. During live streaming shopping, streamers can help me define my needs for the products I buy
	7. During live streaming shopping, streamers can help me determine which products are best for my needs
	8. During live streaming shopping, streamers can provide personalized product customization for me according to my needs
Visibility (Dong and Wang, 2018)	9. During live streaming shopping, I can see detailed pictures of the product
	10. During live streaming shopping, I can see the attributes of the product
	11. During live streaming shopping, I can see a demonstration of how the product is used
	12. During live streaming shopping, I was able to see the products more visually displayed, just as we would in real life shopping
Media richness (Shao et al., 2020)	13. I can use a variety of media (text, pictures, video) to share the information I get from live streaming shopping
	14. I can use small videos to share the information I get from live streaming shopping
	15. I can use a variety of emojis to express my views on live streaming shopping
Attraction (Xu et al., 2020)	16. I think the streamers in live streaming shopping are very talented
	17. I think streamers have a pleasant live streaming style in live streaming shopping
	18. I think the personality of streamers in live streaming shopping is very interesting
	19. I think the image of streamers in live streaming shopping is very attractive to me
Cognitive assimilation (Xu et al., 2020)	20. During live streaming shopping, my existing understanding of the product/service may be affected by the streamer
	21. During live streaming shopping, my current perception of the product/brand may be affected by the streamer
	22. During live streaming shopping, my perception of product value may be affected by the live streaming environment
	23. During live streaming shopping, through communication and interaction with streamers, my preference for products may be affected
Purchase intention (Dong and Wang, 2018)	24. I will use live streaming shopping in the future
	25. I will definitely use live streaming shopping in the next 3 months
	26. I will continue to buy the same goods on the live streaming shopping platform as last time
